# Young Women's Silence and Voice in the Context of Male-Perpetrated Violence

**DOI:** 10.1177/10778012241236673

**Published:** 2024-03-12

**Authors:** Tanja Samardzic, Paula C. Barata, Mavis Morton, Jeffery Yen

**Affiliations:** 3653 University of Guelph, Guelph, ON, Canada

**Keywords:** self-silencing, intimate partner violence, young women, intimate relationships, voice

## Abstract

Our work examines when young women who have experienced abuse choose to (not) speak out and what creates the conditions for (not) doing so. We interviewed 17 heterosexual, partnered women aged 18 to 24 who had experienced intimate partner violence. Many linked silence with fear/anxiety and understood speaking out as a threat to their relationship, while others spoke up despite possible consequences and called their partners out. Some experienced fear at the thought of speaking out but did so anyway. This study nuances understandings of silencing in the abuse context and may contribute to youth programming concerning decisions around (not) speaking out.

“…the secrets of the female adolescent pertain to silencing of her own voice, a silencing enforced by the wish not to hurt others, but also by the fear that, in speaking, her voice will not be heard” – Carol Gilligan (1982, p. 51)

## Silencing in Relationships as a Gendered Practice

Early on, girls are said to become aware of expectations about how they “should” be, including being nice and quiet (e.g., Gavey, 2019), which often conflicts with having a “voice.” Voice has been conceptualized as “having the ability, the means, and the right to express oneself, one's mind, and one's will. If an individual does not have these abilities, means, or rights [they are] silent” (Reinharz, 1994, p. 180). Girls who do not upset others and who do not speak up (i.e., do not have a voice) have been described as desirable, feminine, and good (e.g., Brown & Gilligan, 1992). Many of these pervasive societal standards are understood as converging around the idea that having intimate relationships is a desired social practice and inherent in ensuring moral standing. As such, maintaining those relationships is seen as fundamental (e.g., Jordan, 1985). By adolescence, girls have been taught the power of silence, which ultimately impacts, limits, and regulates their agency. Agency has been globally conceptualized by Emirbayer and Mische (1998, p. 963) as a “…temporally embedded process of social engagement, informed by the past (in its habitual aspect), but also oriented toward the future (as a capacity to imagine alternative possibilities) and toward the present (as a capacity to contextualize past habits and future projects within the contingencies of the moment).” In this manuscript, when referring to agency within the context of intimate relationships, we conceptualize it as girls’ and women's relational autonomy (Mackenzie & Stoljar, 2000). A useful metaphor suggested by Jack (1991) for understanding silencing in the relational context is the idea of not “rocking the boat” or not creating waves that may disrupt the security of the relationship.

Through Jack's (1991) interviews with clinically depressed women emerged “self-silencing,” a construct said to be based on gender-specific schemas that serve as guides for women's social behavior. Jack's (1991, 1999) and colleagues’ (e.g., Jack & Ali, 2010) conceptualization of this construct was largely as a harmful way of navigating relationships while entrenched in shame and anger over their own entrapment and self-betrayal. That was in the same vein as also longing for a relationship and being concerned with relationship maintenance for societal acceptance, moral standing, well-being, and safety. Women are said to employ various silencing-type behaviors, namely (a) assessing oneself through others’ standards (Externalized Self-Perception); (b) over-caring for their partner while simultaneously sacrificing their own needs (Care as Self-Sacrifice); (c) actively inhibiting their self-expressions and actions for fear of conflict, retaliation, and/or relationship loss (Silencing the Self); and (d) an experienced division between one's outer societally “acceptable” version of self—molded by societal and gendered standards of normative femininity—and their inner self that longs to speak out (Divided Self; Jack & Ali, 2010; Towns & Adams, 2015). Within this framing, silencing is not seen as strategic or beneficial beyond a few exceptions (e.g., Impett et al.'s [2005]  findings that self-sacrifice, when done with approach motives, has increased well-being, interdependence, and trust between partners; Woodley's [2019] findings that silence helps enhance religiosity, spirituality, and relationship quality). Indeed, silence has been primarily understood as being all-encompassing and inherently harmful for women and their intimate relationships.

## Self-Restriction in Relationships With Abusive Men

Silencing has been primarily constructed as a pathological practice, which often causes detriment in relationships (e.g., Jack, 1991). There has been little space for considering other constructions, such as the usefulness of silence for maintaining one's safety in relationships where intimate partner violence (IPV) is present. IPV is a fundamental human rights violation and includes any physical, sexual, and/or psychological and emotional harm, including coercive control, inflicted by an intimate partner (Smith et al., 2018). Despite increased legal, activist, and research efforts to combat IPV, prevalence rates remain high. Also, young women under aged 25 remain at the highest risk of experiencing IPV (e.g., Kennedy et al., 2018), with rates during that time ranging between 13% to 40% (Kann et al., 2014; Savage, 2021). It is not surprising that young women are at a higher risk of experiencing IPV than older women given that the period before age 25 is a time when they experience immense pressure to date and have intimate relationships with men (e.g., Tolman et al., 2016). Amid this growing problem, little is known about the way(s) that silencing is manifested in the relationships of young women experiencing IPV.

Scholars have offered different conceptualizations of silencing and its associated function(s) in women's relationships. Thompson et al. (2001) conceptualized silencing as a situational strategy for attenuating future criticality and possible IPV while Whiffen et al. (2007) understood silencing as a more global way of coping with marital dissatisfaction regardless of the conflict present in the relationship. Both presupposed that silencing was something that was “done” in a particular context and that the origins of such behavior came from specific motivations. Others (e.g., Rowe & Malhotra, 2013) took a more constructionist view and understood silencing and women's agency to lie at the intersections of gender, male power, violence, and other factors like intersecting identities, which suggests a multiplicity of meanings and purposes of silence (also see Presser, 2022). It is also important to understand silence in relation to performance because challenges to the status quo can be enacted without self-expression (Hutchings, 2018). While much of the literature on silence is at least a decade old, there have been some recent examples that consider “the unsaid” and “the unsayable” in contexts including and beyond relationships, like medicine and the legal system (e.g., sexual violence by an individual in power; see compilation of chapters by Murray & Durrheim, 2021).

What has received less scholarly attention is that women's experiences of silencing are embedded within societal discourses governing how they “should” be, particularly in intimate relationships. Women's behaviors are understood to be governed by societal expectations and standards that attribute silence and self-sacrifice to femininity and attractiveness (e.g., Brown & Gilligan, 1992; Gilligan, 1982; Weedon, 1987). These standards derive from discourses that are so omnipresent that recognizing them and narrowing down their source(s) is difficult. Recent research offers evidence that young women still experience an inability to speak out because they operate under these societal discourses. Recently, Samardzic et al.'s (2023) participants demonstrated a shared recognition of the expectation that young women engage in silencing behaviors as a way of doing intimate interactions with men. This was especially the case early on in relationships, where they were expected to be “cool” to maintain relationships.

## The Connection Between Silence and Violence

Despite the widespread impact of silencing, especially amid IPV, scant especially recent qualitative inquiry exists. Although scholars have acknowledged silencing as a valid area of study and have offered understanding of it in various contexts, comparatively little work concerns relationships where male perpetrated IPV has occurred. Belenky et al. (1986) found that the women who did not feel that they had a voice with which to speak out in their relationships often experienced IPV. Many of Jack's (1991) participants detailed physical abuse experiences from their husbands and how that contributed to their self-silencing. A prominent theme from Queen et al.'s (2009) interviews with adult women who had experienced emotional IPV was fixing, which represented mental and emotional strategies to “…lessen, cope with, and avoid emotional abuse…” (p. 241). A more recent study of X (previously Twitter) tweets using #WhyIStayed illuminated survivors’ experiences of fear and gender-linked power as important factors in their silence with their abusers (Weathers et al., 2016). Though contributing important research insights, none of these studies included the experiences of young women.

## Current Study

Gilligan (2018) suggested that the #MeToo and #TimesUp movements have allowed women to break the(ir) silence and use their voices more publicly. But what remains unclear are the contexts in which young women who have experienced IPV choose to silence themselves in relationships and under what conditions they choose to speak out. Thus, we explored young women's silencing and speaking out in intimate relationships with men who have perpetrated IPV against them. We aimed to understand (a) how silencing and other self-expressions are experienced by young women in relationships with men who have enacted abuse against them; and (b) how young women challenge or resist the need to engage in silencing within those relationships.

## Method

### Procedure

We received research ethics approval (REB#21-10-006) to recruit young, heterosexual women aged 17 to 24 years who studied and/or resided in Ontario, Canada and who have been in an intimate relationship with a man for at least 6 months to participate in an online survey (the first of a two-part study) about experiences of conflict in their intimate relationships. Recruitment efforts included Facebook and Instagram (e.g., paid ads, groups; 29%; *n *= 45), word-of-mouth (12%; *n *= 19), and eventually, emails to students at select Ontario post-secondary institutions where we obtained additional ethics approval (59%; *n* = 91). Participants completed the following measures, which are briefly discussed given their importance to interview selection: (a) the Conflict in Adolescent Dating Relationships Inventory (CADRI; Wolfe et al., 2001), a 35-item self-report measure of IPV behavior experiences among adolescent dating partners (i.e., verbal/emotional, relational, physical, and sexual abuse, and threatening behavior); (b) the Silencing the Self Scale (Jack & Dill, 1992), a 31-item self-report measure of behaviors that involve self-restriction and over-caring for a partner in relationships; (c) the Communications Pattern Questionnaire (e.g., Christensen & Shenk, 1991), a 35-item measure of communication styles through the process of conflict resolution; (d) three sexual compliance questions (e.g., Gentzler & Kerns, 2004) that inquired about frequency of engagement in undesired sex; and (e) five open-ended questions that we created based on previous literature for more contextual information about (not) silencing in relationships.

At the end of the survey (*N* = 154 completed at least some of it), participants were asked if they wished to be contacted for an interview. More women expressed interest in an interview and had not experienced IPV (29%) compared to those who did not express interest in an interview (24%). Selection happened on a rolling basis as women completed the survey. Only those who agreed to be contacted were considered (36%; *n* = 55). Of those, we invited anyone who met the following IPV criteria (*n* = 39): 1 (*Seldom*) or higher on any of the relational, physical, sexual, and/or threatening behavior CADRI subscales and/or 2 (*Sometimes*) or higher on the verbal/emotional subscale, which ranges from 0 (*Never*)-3(*Always*) (Samardzic, 2019; Wolfe et al., 2001). We sent out 29 invitations in early 2022 and 17 (59%) agreed to participate.

The interviews were done by phone (47%; *n* = 8), a Microsoft Teams video call (35%; *n* = 6), or an in-person meeting in a private university laboratory (18%; *n* = 3) depending on participants’ preference. Participants were sent the consent form, interview questions, and a list of province-wide community resources in advance by TS, as she conducted all the interviews. TS went through the consent process in detail and obtained participants’ verbal consent, then had them select a unique pseudonym for the study. Women who participated virtually or via phone were also briefed on the interview termination protocol, which helped ensure that they were safe and comfortable during the call. If anyone came home, participants would say the word or phrase they had selected at the beginning of our call (e.g., “Bye mom!”) and TS knew to hang up and follow up via email to reschedule the call if specified.

The goal of the interviews was to understand the experiences of silencing and deciding not to silence among young women who had experienced IPV. The questions concerned conflict navigation and resolution with their partner, but generally focused on instances of silencing and related behaviors. Participants were asked about times when they employed strategies to resolve conflict, times when they chose (not) to over-prioritize their male partner's needs over their own, times when they chose (not) to “rock the boat,” and contending with relational expectations (see Appendix for our interview guide). Participants chose between a $20 e-transfer or a $20 e-gift card of their choice (e.g., Amazon). TS then followed up via email to thank them for participating and to re-send the resources and a document detailing how to delete their browsing history on multiple devices.

### Participants

Participants were 17 women aged 18 to 24 (*M* = 21.7, *SD* = 1.8) who had been in a relationship with their current male partner for at least 6 months (*M* = 2.9 years, *SD* = 1.7). Detailed demographic information by participants can be found in Table 1. All participants experienced at least one partner-perpetrated abusive act per Wolfe et al.'s (2001) measure: 88% (*n* = 15) verbal/emotional abuse, 18% (*n* = 3) relational abuse, 12% (*n* = 2) threatening behavior, 12% (*n *= 2) physical abuse, and 53% (*n* = 9) sexual abuse. Also, 88% (*n* = 15) reported having complied with unwanted sex in their current relationship.

**Table 1.** Interview Participants’ Demographic Information (*N* = 17).

ID	Age	Ethnicity	Student	Year of study	Living situation	Employment	Relationship length (years)	Relationship description	# IPV (survey)	Relationship description (interview)
Rose	21	White or European-Canadian	Y	Third	With roommates off campus	Student	2.3	Dating but not living together	2	Boyfriend is very supportive and they have very little conflict
Emily	19	White or European-Canadian	Y	Second	With intimate partner	Student	1.6	Dating and living together	8	Boyfriend gets angry and can sometimes be manipulative in conflict
Robyn	21	Mixed Race	Y	Fourth	With roommates and intimate partner	Employed part-time and student	3	Dating and living together	3	Distrusting and feels betrayed by him but did not want to lose him
Sarah	22	White or European-Canadian	N	–	With roommates off campus	Employed part-time	1	Dating but not living together	2	Wishes she was more independent and thinks that they are not on the same page with their futures
Alex	21	White or European-Canadian	Y	Third	With roommates off campus	Student	0.8	Dating but not living together	12	Referred to his behaviors as “mental abuse” and to their relationship as “previously toxic”
Hayden	22	White or European-Canadian	N	–	With intimate partner	Unable to work	5	Married or common-law	12	Hurts her by throwing personal things (e.g., her weight) in her face during conflict
Zoe	24	East Asian or Pacific Islander or Asian-Canadian	Y	Master's	With intimate partner	Employed part-time	1.9	Dating and living together	4	He demands they keep fighting and expects her to put off commitments to resolve things
Autumn	21	White or European-Canadian	Y	Third	With roommates off campus	Student	4.7	Dating but not living together	1	She is the more reactive one with more things to work on than her boyfriend
Sam	24	White or European-Canadian	Y	Master's	With intimate partner	Employed full-time	4.8	Engaged	4	Her fiancé can be manipulative in conflict
Bridget	23	White or European-Canadian	Y	First	With roommates off campus	Student	3	Dating but not living together	1	They do not have much conflict like in her previous relationship, though some topics are best left untouched
Jane Doe	21	White or European-Canadian	Y	Fourth	With roommates off campus	Employed part-time	1.7	Dating but not living together	2	They miscommunicate and both do not speak up
Stephanie	24	White or European-Canadian	Y	Master's	With intimate partner	Employed part-time	5.8	Dating and living together	1	He takes her speaking up as her blaming him and “blows up”
Jennifer	18	White or European-Canadian	Y	First	With parents, relatives, or guardians	Employed part-time	1.4	Dating but not living together	2	He comes from a broken home and initially is angry in conflict but eventually calms down
Mila	20	Middle Eastern or Middle Eastern Canadian	Y	Third	With parents, relatives, or guardians	Employed part-time	2.2	Dating but not living together	11	Questioned whether her partner's behaviors constituted verbal IPV
Seven	22	Middle Eastern or Middle Eastern Canadian	Y	Fourth	With parents, relatives, or guardians	Employed part-time	3.3	Dating and living together	3	Has never worried about him abusing her as he is a “very good man”
Taylor	21	White or European-Canadian	Y	Third	With roommates off campus	Student	1.4	Engaged	4	He gets set off by little things but the anger is not directed toward her
Katie	24	White or European-Canadian	Y	Fourth	With intimate partner	Employed part-time and student	5.8	Dating and living together	2	He gets angry and cannot listen, but the anger is not directed at her

Despite following clear IPV criteria, there was great variability in participants’ qualitative descriptions of IPV within their relationships. Some (e.g., Alex) named experiences as abusive, some (e.g., Mila, Hayden) detailed problematic experiences more euphemistically, and others (e.g., Seven) specified that their partner was not abusive. But we resisted clustering participants in any such way for two reasons. First, we classified their experiences as “abusive” with limited information (e.g., answers to de-contextualized survey questions), thus lacking valuable contextual information (e.g., both partners have attended therapy since she filled out the survey). The interviews allowed women to provide details about the conditions within their relationships in their own words at that point in time, and we aimed to stay true to those experiences. Second, we recognize the contradictions and tensions inherent in navigating intimate relationships and understand that women may align themselves with more than one way of thinking (e.g., Weedon, 1987).

### Theoretical Lens and Analytic Strategy

Amid criticisms that the highly used thematic analytic approach is overused, unreflexive, and conceptually confusing, Braun and Clarke (2019) produced a rethought-out approach that accounts for the researcher's position and allows the construction of and guidance through a story within the data (e.g., Braun et al., 2016). The reflexive thematic analysis approach calls upon researchers to be thoughtful and deliberate in their methods and to grapple with research assumptions (Byrne, 2022). In using this approach, we could locate participants within the larger sociopolitical/cultural context post #MeToo and #TimesUp (Braun & Clarke, 2019). We also considered our influences on the research, which are discussed further in the Reflexive Approach section below.

The data were rich and provided us with a well-rounded picture of the ways that young women navigate their intimate relationships. TS manually transcribed almost all the interviews, receiving minor assistance from two undergraduate research assistants. This allowed her to familiarize herself with the data. She read each transcript in its entirety to further understand each participant's unique experiences, then re-read the transcripts, noting anything common between participants as well as places where there were deviations. Those notes formed our initial codes, documented in NVivo12, which set the foundation for theme generation. Our focus shifted from individual information to more aggregated meaning across the dataset. Next, it was important to review the proposed (sub)themes and corroborate each with sufficient data, then name each. We used exemplar quotes for each (sub)theme in our Findings section to help illustrate them more clearly.

### Our Reflexive Approach

We have grappled with our positionality from the conception of TS's dissertation idea. TS is a White, heterosexual, cisgender, first-generation Canadian woman whose feminist approach has adopted a critical component to deconstruct the laden expectations of (young) women. The other authors served as her dissertation committee, and each brings important expertise to this work. PB was TS's Ph.D. advisor and her explicitly feminist work centers around IPV and sexual assault. The other authors were TS's committee members: MM's work focuses on social justice issues, specifically gender-based violence and critical community-engaged scholarship and JY has expertise in several qualitative methods and is attentive to important theoretical considerations. We acknowledge that culture and class locations such as ours shape academic work and that our focus and the experiences that were raised in this study may be more familiar to White, Western, heterosexual, cisgender, middle-class women than to women occupying other intersecting social locations.

## Findings

We generated two higher-order themes, one with three subordinate and the other with two subordinate themes, about navigating silence within intimate relationships. In the first theme, we highlight the entanglement of silence with fear and anxiety that came through in several experiences of being in relationships with abusive men. And despite inquiring almost exclusively about silencing, several young women's answers reflected instances of speaking out. Those experiences are captured in the second higher-order theme, where they used their voice and spoke up despite the potential for consequences (e.g., their partner's anger, relationship loss). Please note that ellipses (…) were used within quotes to indicate that a chunk of text irrelevant to the analysis was removed and dashes (—) were used to demonstrate that the next quote reflects a new interview.

### Theme 1: The Entanglement of Silence and Fear/Anxiety

“I would feel like I’m walking on glass”

While reflecting on conflict in their intimate relationships, participants explicitly linked silence with fear and anxiety. Participants noted that the fear of them saying something wrong and possibly upsetting their partner necessitated silence. Two such examples are Hayden's (22, White, partnered for 5 years) and Emily's (19, White, partnered for 1.5 years):

Hayden: if something bothers me, I keep it to myself. We’ve been together for 5 years, so I learned things that upset him, and I try not to ensue them. He doesn’t like if I take nap[s], so I don’t, even if I stay up with the baby. I feel like I’m walking on eggshells.

Emily: sometimes I would feel like I’m walking on glass. If I say the wrong thing, it’ll set him off and make him mad. Even the slightest joke… I’m scared to say something wrong.

Hayden's use of “walking on eggshells” and Emily's use of “walking on glass” are visceral depictions of two important factors. The first is the nature of their relationships, which include varying degrees of IPV. The second is the “mental work” inherent in their attempts to keep things running smoothly. We use this term to describe women's attempts at both internally assessing whether to speak up (especially given previous evidence that speaking up would be poorly received by their partner) as well as carefully crafting how they say something. Mental work was also referenced by Jane Doe (21, White, partnered just over 1.5 years), who eloquently described the labor-intensiveness inherent in this concept:

I was always really worried about saying something wrong because that's what typically would happen in my past relationship. I would think about how I’m going to word things, so I always need that time to think how am I gonna word this without upsetting him.

TS: Right, so a lot of that mental energy.

Jane Doe: I need that time. Yeah.

TS: So, it was sort of a lead-up for you where you’re like this is bothering me, I gotta wordsmith this in my head, and then I’ll bring it up.

Jane Doe: Yeah, pretty much.

Their enactment of silence was strategic. Taking additional time to formulate the right words to avoid “saying something wrong” was established as protective. Employing this protective strategy allowed Jane Doe to avoid the possibility of upsetting her partner and creating and/or escalating conflict. The fear and anxiety were coupled with silence, which was understood as necessary to avoid losing their partner (e.g., “He made me feel like I can’t lose him. What would I do? My world would be upside down” [Alex, 21, White, partnered for just under 1 year]; “I feel a bit anxious because I’m afraid of making him upset and putting our relationship at risk” [Jennifer, 18, White, partnered just under 1.5 years]). Silence is important in abusive contexts, where not only is it a way of maintaining peace but it may also function as a way of keeping them safe and maintaining the integrity of their relationships. But the broader undertone of fear and anxiety that came up for participants appeared in three different ways in the data, discussed next.

#### Subtheme 1a: Taking Responsibility for Him and the Harmony of the Relationship

“I felt it was my job to clean up the mess”

Participants’ discussions about needing to take responsibility for their partner often included over-prioritization and shouldering the burden of his actions, especially with others. Alex's (21, White, partnered for just under 1 year) partner's drinking necessitated this:

When he was drinking, I felt it was my job to clean up the mess. I felt like as his girlfriend, it was my job and I had to go ease the situation. I had to take him to bed and apologize on his behalf. So many times, I’d have to drag him up the stairs, lock him in the room, and I’d have to go downstairs and apologize to whoever he said something rude to. I guess I’m supposed to fix him [and] to deal with it… The next day when people would be mad at him, it was always my fault. “Why would you let me do that?”

There was a relational element to Alex's care, that is, her ensuring that his reputation remained intact amid his belligerent behavior with others. In this situation, she was blamed for *not* keeping *him* silent. Ensuring that her partner's reputation remained unscathed required Alex to ignore the feelings of hurt and disappointment as well as her idea of “proper” relationships more generally.

Participants also described instances of needing to take ownership and responsibility for the conflict, including for their partner's role in it. One example is Stephanie (24, White, partnered for almost 6 years), who discussed taking responsibility amid her fiancé's criticality:

I’ve tried to be aware to apologize and take ownership of any mistakes, whether I believe that that's actually a mistake that I made or not. I would rather apologize because it's not worth it. So, I’ll apologize, acknowledge I did something to upset him, reduce the amount of explaining because he interpreted it as excuses, [and] acknowledge how he perceives it… I wouldn’t necessarily feel like whatever happened or was said was wrong. Maybe it was. It could have been approached better or I could have said something different[ly]…

The focus and responsibility turned away from the male partners and onto the young women. Both above extracts reflect a tension regarding the need to take responsibility while also knowing that it was not theirs to bear. In Stephanie's instance specifically, her fiancé's push for her to look inward reflects common relationship communication advice (i.e., “I’ll apologize, acknowledge I did something to upset him…”) that may have seemed more familiar to her regarding how conflict is resolved in intimate relationships and thus may not have been a point of contention for her. Stephanie's disagreement about “…whatever happened or was said was wrong” was something she kept to herself, and she instead apologized and supported his version of events. The silencing came from the onus being on Stephanie. Such a situation may breed doubt in the women, destabilize their confidence over time, and perhaps work to reinforce their silence.

#### Subtheme 1b: Justifying His Anger

“The aggression they’re putting behind the words isn’t about you”

Some young women talked about needing to manage their partner's anger, which seemed to be a way to contend with his problematic behavior toward them. They provided multiple reasons for their partner's anger, like that “he didn’t know how to communicate properly” (Seven, 22, Middle Eastern, partnered for just over 3 years) or “in the past, he had been hurt” (Mila, 20, Middle Eastern, partnered for just over 2 years). Katie (24, White, partnered for just under 6 years) provided a more detailed account of reframing and managing her boyfriend's temper:

Sometimes I have to put on kid gloves. If he's getting riled up, I have to bring him down… It's not an upset where he's aggressive, which would obviously be worse. It's an upset where he doesn’t know where to put that emotion and as soon as he feels cornered, “I have to fight.” I know that's from his childhood. Doesn’t excuse it but he's just angry and oftentimes I can honestly tell that the anger isn’t even towards me.

Katie's description of needing to de-escalate him after he reacted angrily, which in this case was a discussion about the inequality of housework responsibilities, included both diminishment and an attribution of his anger as being a by-product of his upbringing. Despite attempting to speak out about her needs, she needed to “put on kid gloves” to “bring him down.” This resulted in a focus on him and consoling him by emphasizing that “this isn’t an argument; I’m just bringing something up” rather than further discussing what she wanted and needed from him. Taylor's (21, White, partnered for just under 1.5 years) experiences were similar in that she also had to put her partner's needs first:

It does make me feel sad and sometimes angry because [the conflict and his reaction to the conflict] ain’t about me so why is it me you’re taking it out on? It takes a lot of strength to re-center and regulate your own emotions and this is where putting my partner's needs above my own has to come into play… [You have] to be able to take that step back and realize the frustration isn’t about me and they’re just heated up and venting. You have to be able to realize the aggression they’re putting behind the words isn’t about you.

The extracts illustrate how Katie and Taylor's justifications and management strategies worked to free their partners of the consequences of their respective actions and the responsibility of needing to remedy the situations. Clear awareness of the anger (e.g., “riled up”) as its underpinnings (e.g., “that's from his childhood”) necessitated a focus on their partner while inhibiting their own needs. Such self-inhibition and over-care for their partner's needs amid his anger worked to reinforce the young women's silence.

#### Subtheme 1c: Speaking Out as a Threat to Relationship Integrity

“If I be direct, is he gonna dip?”

Embedded in several accounts was a fear of risking the viability of the relationship if they spoke out about what they wanted to their partner:

Bridget (23, White, partnered for 3 years): We don’t have productive conversations on future stuff because we’re not in a place where we’re thinking about marriage or kids, but I think it's always at the back of our mind that I probably want kids and he probably doesn’t. If it ever come[s] up, I’m hoping he’ll say something he never will… His past relationship ended because she was ready to move in with him and he was not. Knowing that that's how his last relationship ended makes me want to bring it up even less…

Knowledge of her partner's hesitance and discomfort in discussing the(ir) future was a powerful reinforcer of Bridget's silence. Despite wanting to engage with him about those topics, she conveyed the necessity of her silence to not push him and to secure the integrity of their relationship. Similarly, Robyn (21, Mixed Race, partnered for 3 years), who at the thought of telling her boyfriend how she felt about him being inappropriately sexual with their roommate, said, “I feel like there's more at stake. If I be really direct, is he gonna dip? It's like, fuck, I did not just go through this whole year of shit for him to leave.” Robyn had previously described a year-long battle with herself about how best to handle the inappropriate “friendship” between her boyfriend and their roommate. She eventually uneasily supported their friendship while internally feeling upset and betrayed when he prioritized the roommate's needs over hers. She did not speak up despite the feelings of betrayal and hurt, and instead kept silent to maintain his comfort. Both extracts demonstrate a fear of losing the relationship if they spoke out.

Other participants’ partners were clearer about the consequences of them speaking out and either threatened to leave the relationship or questioned why they stayed with their male partners.

Alex (21, White, partnered for just under 1 year): Most of the time, I wouldn’t even bring [his drinking] up because I didn’t want the arguments [or to] be more upset. When I would, I would say how I was feeling, and he often would hit me with the “then leave me. Break up with me. If you don’t like the way I am, leave.” I don’t want him to leave me so OK, I’ll suck it up. I would put my feelings aside. I won’t bring it up again.

Over time, feeling unheard by him was a powerful silencer for Alex and her not speaking up ensured that he would not threaten their relationship. In the face of such powerful threats, for many of our participants, silently coping with problematic behavior was preferable to losing the relationship.

### Theme 2: Speaking Up Despite Possible Consequences

“If something is bothering me, I will let him know”

Despite most of our questions concerning silencing, a surprising proportion of young women emphasized using their voice and speaking up as an important feature of their relationship. Many also were willing to speak up, some even with righteous indignation, despite possible consequences (e.g., their partner's anger, a relationship breakdown, further IPV inflicted upon them). Participants emphasized that “conflict is good [and] healthy” (Seven, 22, Middle Eastern, partnered for just over 3 years) and that “[they’ve] been trying to bring [concerns] up as soon as they occur” (Jennifer, 18, White, partnered just under 1.5 years). Some, such as Rose (21, White, partnered for just over 2 years), emphasized the importance of communicating:

Communication, definitely number one. We’re both very comfortable in this relationship. We know that we can say anything to each other and not have any negative judgment or connotation to it. If something is bothering me, I will let him know and we can work together to find a solution and same for him…

Sam (24, White, partnered for just under 5 years) also highlighted the importance of communication and how it can be bettered with the skills learned in therapy:

Recently, I’ve attended therapy and I’ve learned better communication skills that I’ve brought into the relationship and talked to him about and he's actually been open… We were in a situation [where he got mad instantly] after I talked to him about tactics I had learned. We used them and the conflict was resolved in a much better way.

Speaking up about relational conflict helped to prevent a build-up of resentment brought on by self-suppression. But what differed between participants who endorsed the practice of speaking up by using their voice as a way of doing relationships was that while they still experienced that fear about doing so, it functioned differently:

TS: would you say where you’ve “rocked the boat,” that his reaction is comparable to when you just bring up something like, “we need to split the housework better?”

Katie (24, White, partnered just under 6 years): When I rock the boat, it's worse, his reactions, ‘cuz usually it's something I’ve been avoiding for good reason [laughs] and is gonna upset him. But I’m like no, I *have* to say this!… I took care of him because that's what I thought was expected of me. It made me feel terrible about myself and it made me start to resent him, almost like he was taking advantage. Eventually I exploded and it didn’t end well because I definitely didn’t communicate what I was feeling very well [laughs]… The biggest time I rocked the boat is when we broke up because I initiated it. I said, “…I want [us] to be happy and this is not working.” Even if we hadn’t gotten back together, it would’ve worked out because we both have to be happy. But it's terrifying to do that when you don’t know how the other person's going to react.

Despite possible consequences, namely her partner getting angry and their relationship ending, both of which happened, Katie still “rocked the boat.” Taylor's (21, White, partnered for just under 1.5 years) experience was similar: “waves happen, but if you’re constantly scared of your relationship falling apart because of something you wanna say, you’ve gotta really think about the relationship.” Taylor's partner reacted with understanding and apology rather than the negativity that she expected, and she saw speaking up as necessary to get to a better place within her relationship: “Your relationship's based on communication and when you can’t communicate, you build resentment… Usually after rocky water come some pretty calm ones.”

We also heard examples of participants having difficult conversations, which sometimes led to temporary breakups. Autumn and Katie experienced breakups while Jane Doe and Alex each threatened to end their respective relationship. Regardless, a concern for the state of both partners’ well-being was central to their speaking out. For instance, Alex's concern was for her partner's well-being as he was battling alcoholism as well as her own mental health while having to provide wraparound care, while Autumn expressed upset about the miscommunications and lack of productive communication with her partner and how harmful that was. Despite everyone experiencing at least one IPV behavior, openly voicing their needs was paramount. This was so much so that some relationships were dissolved, indicating a stronger emphasis on both parties’ well-being over having a relationship where there was little communication and/or satisfaction. The way that young women conceptualized speaking out was two-fold and is discussed next.

#### Subtheme 2a: Calling Out Gendered Expectations

“I feel like I overwork myself and I’m underappreciated”

A commonality among several participants was being more expressive about their needs, particularly when it came to gendered expectations of them. Disputes over household responsibilities were especially common given that many were cohabitating with their partners. Young women described working just as much as their partners, though the domestic duties still often fell on them. Many were therefore quick to voice their displeasure at such inequality:

Seven (22, Middle Eastern, partnered just over 3 years): I’ve said, “I do the laundry but also do the dishes, so you have to choose one.” And he’ll be like, “why do I have to choose? I cook.” “Good for you! Anytime I cook, I do the dishes so how about you [do both]? I’ll do the laundry [and] clean.” And then we’ll get into an argument. He's like, “why do we always have to go through this?” and I’m like, “’cuz I overwork myself and I’m underappreciated.” He's like, “I appreciate you.” I’m like, “you don’t show it.”

These women resisted the expectations that they do all the housework and instead openly called for equal responsibility. Participants seemingly lacked concern for their partner's reaction to those requests, and this was also described in other ways. Autumn (21, White, partnered for just over 4.5 years), in response to her boyfriend contradicting her beliefs while she was “ranting” to him, said that “I just want you to listen and be there for me if I’m frustrated or upset. No offence but I didn’t ask for your opinion.” She identified herself as being more reactive and her boyfriend as calmer, which likely afforded her greater comfort in speaking out. What was important was their ability to speak out without fear, as described earlier.

Some men were not as receptive to their female partners speaking out about the inequality concerning gendered expectations. Stephanie's (24, White, partnered for almost 6 years) fiancé got defensive when she requested his assistance with meal preparations for their household, something she usually did in addition to completing her graduate school and work-related responsibilities:

He blew up and was like, “if you don’t want to help [meal plan and write a grocery list], then I’m going to do my own.” And I’m like, “that doesn’t make sense. We live together.”… He walked out and needed to cool off because I was asking too much of him.

Katie's (24, White, partnered just under 6 years) partner invoked dated notions of heteronormative gender roles about men's role in a household being financial. When she pointed out that her work and school amounted to an equal amount of time as his full-time job, a confrontation ensued where she explained the injustice, and highlighted that when he lived alone, he paid the bills and did the housework. In response to how it felt telling her partner how she felt, Katie explained the following:

It felt a little nerve-wracking but good to say. It felt good to just get it out and be like, “listen, this isn’t fair.” In so many ways, he is an incredible partner, but when it comes to that, it's been a struggle. That's why I wanted to make him understand that I’m not saying you’re a bad person. I’m just saying that you’re doing something that is harming me and [will] continue to harm me for the rest of our relationship if we don’t deal with it.

She aimed to dismantle his gender stereotypical ideas, many of which were ingrained via the gendered messages that he internalized through his upbringing. She also highlighted his subconscious thinking and how difficult it can be to undo that, but because it was harming her, she needed to.

Amid those negative reactions, participants were able to object to outdated notions of women needing to do all the domestic work and also prioritize their own needs. This is important because all our participants had experienced at least some IPV, yet they appeared to be confident in being outspoken. Doing so helped these participants to counteract silencing and harmful gendered norms (e.g., Katie telling her partner that his buy in to gendered stereotypes of heterosexual relationships was harmful to her and she finally told him it was “unfair,” which he responded to with understanding; Seven emphasizing that she felt underappreciated and overworked to her partner, who then picked up more of the domestic duties).

#### Subtheme 2b: Calling Out Inappropriate Behaviors That Worked to Silence Them

“I didn’t appreciate how you spoke to me”

Some participants called out behaviors that they perceived as inappropriate within their relationship and that functioned to silence them and did so in the face of their partner's negative reaction. Emily (19, White, partnered for 1.5 years) detailed experiences with her boyfriend earlier on in their relationship: “when we started dating, [he said] rude things. I don’t know if he tried to put me down. I’ve come out now and said, ‘those things hurt my feelings. You shouldn’t say them.’” Her willingness to oppose him and tell him that the things he was saying to her were hurtful was not immediately apparent until this explanation:

He would stay at my house and get mad and leave. I’d have to call and beg him to come back. He wanted a reaction [and] got mad to know that I cared. So, I caught on. One time, he said, “I don’t know if we’re gonna work out” and I was like, “OK.” He was like, “you’re gonna let me go?” I was like, “I know that you’re not going to” and he was like, “so, you’re walking all over me?” “No, you’re the one telling me that you’re gonna [leave]. I’m saying OK ‘cuz I know you won’t. You’re trying to get a reaction.”

Emily had previously described feeling like she was “walking on glass” (see Theme 1) and indicated how his put-downs worked to ensure that she complied with his wants. Despite these circumstances and his anger, she still proceeded to call out his (empty) threats. Seven (22, Middle Eastern, partnered for just over 3 years) also emphasized her displeasure with her boyfriend's temper:

He had a really short temper at the beginning of our relationship where if I said anything, he would snap at me, and I would be upset because “why did you snap at me? I’m telling you how I feel.” He didn’t know how to communicate properly. Now, I tell him how I feel. He tells me ways that he’ll fix it, apologizes, acknowledges my feelings, and fixes it.

TS: How did that change for you both?

Seven: We would reflect on our relationship, and I used to tell him, “I don’t like your short temper. In a second, you can pop off. When I’m telling you how I feel, I feel like I’m wrong because my feelings are valid.” The fact that I’m telling him how I feel is already hard enough and then he would lash out and I would be like, oh my gosh, why did I do that? After a while, I was like, F this, I’m telling you how I feel.

TS: You said, “why did I say that?” How would you describe that? Regret? Guilt? Fear?

Seven: Definitely not fear. Probably guilt. Why did I do that when I could’ve just kept my mouth shut? Now, I don’t care how you feel. You’re gonna fix it or we’re not chatting.

Seven clarified that her second-guessing about speaking out was rooted in a concern for her partner's feelings. She understood that expressing her feelings would upset him, so she instead suppressed herself (“he would lash out… why did I do that?”). She specified that his anger came from him not knowing how to communicate “properly,” so these conversations helped her teach him to communicate better. Seven noted that the metamorphosis to a place of comfort with and an emphasis on being expressive regardless of his proclivity to “pop off” came from having deep and meaningful conversations together. Those conversations helped her partner to reflect on his previous ways of dealing with conflict as not being productive or sustainable. Others (e.g., Jane Doe, Sam) reported a similar shift as well.

While some participants’ verbalization of disapproval concerning their partner's reaction was instant, for others, it was not always immediate and instead took time.

Stephanie (24, White, partnered for almost 6 years): I asked him if there was a certain accommodation that we can make [at our shared workplace] to serve a customer in a wheelchair, which wasn’t the protocol, but I asked to advocate for [them]. He told me, “you know this is not protocol. Why would you bother asking?” Usually, I would have not said anything [and] sucked it up, but this time, I sent him a text and said, “I know that because I’m your girlfriend, you felt comfortable saying that, but I didn’t appreciate how you spoke to me.”

Stephanie's go-to reaction was to silence herself, something that her fiancé likely expected in this situation too, but she eventually called him out for his inappropriate behavior and hostile tone toward her. Like Emily above, Stephanie's fears were also reflected in the first theme. This situation, however, played out differently:

My default would have been to accept that [he did not want to talk for hours after a conflict] and to demand that we talk, but I did because I’m not going to sleep. I’ve waited a reasonable amount of time. We should be able to have a conversation. He not only didn’t want to speak, but he left town without telling me and went home to his parents.

Stephanie's experiences of being cheated on by her previous partner colored her preference for tiptoeing around conflict. But despite her fiancé's avoidance after his anger toward her and worries about “stirring the pot,” she still felt secure enough to challenge him.

## Discussion

We sought to examine young women's experiences of silencing while in relationships with men who had engaged in IPV against them. We saw two different ways that silencing was discussed. Some detailed the strategies that they employed, namely justifying their partner's anger and taking responsibility for him, to cope with relational conflict. Here, speaking out was seen as a threat to the integrity of their relationship and was often coupled with feelings of fear. Some others, in response to questions about silencing, retorted that instead, they saw the act of speaking up to be important, despite the possible consequences they could face. Those women exhibited confidence when calling their partners out for unacceptable behaviors and adhering to dated gendered expectations, irrespective of the varying levels of abuse that they had experienced. There were some participants whose experiences included both a fear of speaking out (e.g., because of the threat of their partner's anger) and instances of doing so anyway despite the various IPV they had experienced. Those instances nuanced the function(s) that silencing plays in the context of IPV as being equally necessary for relationship maintenance and not “rocking the boat” while also necessary for achieving authenticity and having their needs met.

Everyone reported experiencing one or more acts of IPV in the online survey; however, many did not self-identify their partner or relationship as such during the interviews. Despite this, we saw parallels between their experiences and those of women in the IPV literature (e.g., Øverlien et al., 2020). Unsurprisingly, far more women indicated experiences of silencing while simultaneously feeling afraid. In the IPV literature, a key feature in women's accounts has been the fear that comes with anticipating the next bout of violence and thus being constantly on guard (Lindgren & Renck, 2008). Also discussed by scholars in this body of literature are descriptions of strategies, like placating him (Goodman et al., 2003), behavioral disengagement (Foster et al., 2015), taking responsibility for his actions (Waltermaurer, 2012), and justifying the abuse (Bauman et al., 2008). These are all strategies that we heard in our participants’ experiences. The parallels between our participants’ experiences with those of other women survivors underscore the importance of silence as a strategy to maintain calmness and safety within intimate relationships.

Most (71%; *n* = 12) emphasized the importance of having an intimate male partner as integral for their well-being and social support. This is likely a powerful motivator in opting to suppress themselves, at least sometimes (e.g., Jack & Ali, 2010). Participants like Hayden and Alex, whose experiences were best captured in the first theme only, reflected those of other (young) women whose narratives have been documented in the existing IPV (e.g., Tuval-Mashiach & Shulman, 2006) and self-silencing (e.g., Shulman et al., 2018) scholarship. They over-prioritized their partners, took responsibility for their partner's actions, inhibited themselves, and to various extents gave up some or all of what they wanted. This is unsurprising given Thiesmeyer's (2003) assertion that while silence is seemingly rooted in personal choice and actions, it is also accompanied by sociopolitical judgments of what is (not) societally acceptable.

Most interesting were the experiences of those who expressed fear about speaking up but who chose to do so anyway with righteous indignation and a lack of concern about possible consequences. The extent of their IPV experiences varied; however, they chose to use their voice for multiple reasons, including a recognition that they too deserved to be heard, and the need to speak up for the good of the relationship. This finding is important when considering the impact of gender and age given that these women are younger and may therefore perceive less of a risk of speaking out compared to older women who may be married, live with, have children with, rely on (e.g., for immigration status), and/or be financially dependent upon their abusive male partner (e.g., Chantler, 2006; Cutter-Wilson & Richmond, 2011; Jack, 1991; Korkmaz, 2021). Ideals about relationship egalitarianism (e.g., Daminger, 2020) were likely contributory to their comfort in speaking out. Important societal changes have likely allowed women to feel entitled to being outspoken in ways that were not available to them in the 1980s/1990s. Participants’ assertions about what they needed and their partners’ reluctance to comply represent evidence of long-standing structures of male dominance being at odds with contemporary beliefs about the progress toward equality (Baker, 2012). As values of patriarchal dominance are said to be more openly questioned, more women likely feel empowered to subvert and resist such male-dominated power systems and thus are able and entitled to speak up (Lewis & Simpson, 2017; Samardzic et al., 2023). Though relationship maintenance remains important, we have provided evidence for young women being willing to prioritize speaking up in certain situations despite potential relational consequences that could be faced.

### Limitations

Our first limitation was our IPV categorization, where more frequently occurring experiences counted and thus indicated having experienced IPV. Such a way of categorizing abuse included young women whose experiences were abusive but who may not label their partners as abusive. However, including these young women's experiences and perspectives allowed for a nuanced understanding of silencing within a myriad of relationships, with key similarities appearing. Future researchers may wish to employ a measure such as the Composite Abuse Scale (Hegarty et al., 1999), which includes four subscales that account for the frequency of experiences. Alternatively, researchers may wish to have young women self-identify/select to participate, though this requires the labelling of their experiences as abusive, which may be difficult (e.g., due to the stigma and shame associated with staying, Enander, 2009) or take women time to process and label. In such a sample, the consequences of giving voice to their feelings may be clearer and the speaking up itself may look different between the two samples. Another alternative is to attend to behaviors that are not typically labelled as abusive (e.g., sporadic put-downs). Finally, researchers may wish to specify the timeframe of occurrence because some of our participants discussed conflict and IPV that occurred earlier in their relationship that no longer applied at the time of the interview, though this was also a strength. After all, it allowed us to hear about how some participants resisted silencing.

Another important limitation was the lack of sample diversity, especially concerning participants’ racial/ethnic backgrounds and student status. Indeed, many (76%; *n* = 13) were White young women, and most (88%; *n* = 15) were students, which was mostly because of our recruitment strategy. We made several attempts to recruit a more diverse sample but had little response. TS did not specifically ask about the influence of culture and ethnicity as it pertained to women's relationships, but two participants invoked gendered stereotypes of the traditional Italian and Muslim women, respectively, when it came to household responsibilities in their relationships. Future researchers should more purposively recruit a more diverse sample of young women and directly inquire about the role of important elements of identity that influence the navigation of intimate heterosexual relationships where IPV has occurred. Connecting with community organizations that work directly with young women ahead of time and using more targeted and personalized recruitment strategies may be fruitful.

### Directions for Future Research

Future researchers may wish to expand upon our findings to explore contemporary elements of silence as well as decision-making around speaking up. One specific concept that was novel and had not emerged through our review of pertinent bodies of literature or in our previous qualitative inquiries with young women was the idea of “mental work,” which may be a different dimension of self-protective silencing. This term may also differ from other similar concepts like emotion work and mental labor. Emotion work has been conceptualized primarily in the workplace context and includes a consideration of the required, felt, and displayed emotions for a given situation (Zapf et al., 2021). Based on the emotional requirements of a given job, employees will then use a host of emotion regulation strategies to manage the experience and expression of their own emotions in that situation (e.g., Gross, 2002). More aligned with our conceptualization of mental work is mental labor, which has been used to reference the cognitive dimension of unpaid work in the context of childcare and housework. It includes cognitive activities like thinking and remembering, is managerial and includes planning and monitoring, is communally oriented and useful to others, has an anticipatory component given that the focus is on the future, and may include elements of invisibility such that it goes unnoticed by others (see Reich-Stiebert et al.'s [2023] review of several related terms; also see Walzer, 1996). Such a definition is comprehensive but may not fully encapsulate what arose in our study, especially given key demographic differences in this sample (e.g., few of our participants were married and/or mothers, many participants’ insistence on gender egalitarianism within their relationship).

In our study, what we term “mental work” involved significant mental effort, including weighing the consequences of speaking out and considering how best to word things in light of their past experiences (e.g., the last time I mentioned this situation, he got angry and left the room). Women's struggles of wanting to be open and honest while also knowing what will happen if they do speak up may put them in a double bind. Double binds in women's lives have been a topic of scholarly focus for decades, including in heterosexual sex (e.g., Muehlenhard & McCoy, 1991) and the workplace (e.g., Shapiro et al., 2008). Exploring the concept of mental work, both by operationally defining it in light of available research on related topics and by exploring its role in creating an additional double bind in women's relationships, is an important next step in this line of inquiry. For instance, women engaging in mental work to figure out whether and how “best” to say something may impact the communication and authenticity within their relationship, yet speaking up without engaging in mental work may bring consequences such as their partner's anger. Such a double bind warrants further exploration, as does looking to situations where women perform significant mental work for fear of their partner's reaction and their partner reacts well and reinforces their use of voice would also be illuminating. Relatedly, understanding more fully under what condition(s) and for what reason(s) women do decide to speak out would be important as we could not explore this in our own work.

Exploring relationship dynamics (namely the IPV context) and having women talk about their decision-making processes, including the mental work that they may do and consideration of other facts that may facilitate and hinder their decision to speak out, could be another line of future inquiry. In our study, fear seemed to be a central deterrent for women speaking up. However, fleshing that out more, including what exactly women fear (e.g., fear of [more severe] violence, fear of being embarrassed) would be a valuable contribution.

### Practice Implications

These findings provide important information for existing dating violence prevention programs for youth, which have proliferated in recent years in (post)secondary and community settings (e.g., Crooks et al., 2019). These findings could be especially useful when educating youth on topics like healthy relationships, conflict resolution, and communication (e.g., Tharp et al., 2011). For instance, learning to notice when and for what reason(s) one is self-suppressing is important to recognize red flags such as silencing because of a fear of one's partner and his actions. These findings may also be useful to practitioners when working with young women currently in relationships with abusive men, regardless of if they label their experiences as such. There are grey areas that come with the label of “abuse,” which makes the definition of boundaries difficult. Providing a more inclusive perspective on what constitutes IPV, like using euphemistic language (e.g., “conflict” or “stress”; Samardzic, 2019) and focusing on more common forms of abuse, may help serve more women as they navigate coping with their partner's behaviors. One-quarter of our sample identified the importance of therapy for their mental health and relationship well-being. Some even described teaching their partners strategies that they learned from therapy, thus demonstrating its value.

### Conclusion

We explored the silencing experiences of young women in relationships with abusive men. Unlike in previous foundational works, our participants’ experiences reflected novel ways of navigating decision-making around when to (not) silence in their relationships. Although some detailed experiences of fear and feeling as though they were walking on eggshells or glass, several of these participants provided vivid examples of challenges to the way that they “should” be. Challenging such dated gendered expectations regarding housework and calling out inappropriate behaviors that worked to silence them suggest that young women speaking out in their intimate relationships is paramount for having their needs met and ensuring equity, despite potential consequences. Our own early conceptions of silence as something that was static, sweeping across the relationship, and inherently negative changed through this research. Based on our compelling findings, future researchers should nuance their understandings of silence to something that may be more fluid, situational, and done intentionally with specific goals in mind. What we ultimately saw was that such challenges to the silencing imperative may reflect societal changes that now empower women to subvert and resist male-dominated systems of power where there is a newfound entitlement to speak up/out.

## Appendix: Interview Guide

1. I would like to start by understanding what being in a relationship and having an intimate male partner means to you. How important would you say this is in your life?
2. Could you describe what conflict in your relationship with your partner looks like?
3. There are lots of ways that people try to resolve conflict when it arises in their relationship. What kinds of things do you do to keep things going smoothly?
  - *[If they provided at least one strategy and also discussed the importance of relationships above]* Can you describe a time when you tried [insert the strategy that they mentioned] and it did not work? How did your partner react?
  - *[If they provided at least one strategy but previously said that relationships were not very important]* You listed a lot of things that you do to keep the relationship running smoothly, but you also noted that relationships are not that important to you. I have noticed this in interviews with other women as well. Can we talk about that? How important is it for you to do these things?
4. *[Asked if they did not mention over-caring as a strategy]* One strategy that we heard other young women in a previous study of mine using was putting aside what they want and need and prioritizing their partner's needs. Can you describe a time when you did this?
  - Can you describe a time when you thought about using this strategy to reduce or avoid conflict, but instead decided not to?
5. *[Asked if they did mention over-caring for their partner as a strategy]* You mentioned putting aside what you want and need and prioritizing your male partner's needs. Can you describe a time when you did this, perhaps during a conflict with him?
  - Can you describe a time when you thought about doing this but decided not to?
6. What does it mean to you to be your true self and speak out about what you want and need in your relationship? How important is this to you
  - Can you describe a time when you wanted to be your true self and speak up about your needs but were unable to in your relationship? Are there any factors that prevented you from doing so?
7. Something we heard before was the idea of “rocking the boat.” You can imagine calm waters when there is no disruption, but when a wave comes, the boat rocks and puts those in it at risk of falling out. In the same way, young women see speaking up as creating waves and possibly upsetting their partners. Can you describe a time where you “rocked the boat,” so to speak? What were you feeling? What was your boyfriend's reaction?
8. Can you describe a time in your current relationship when you felt like you had to do what was expected of you?
  - What do you think your partner's reaction would have been if you had not done what was expected at that moment?
9. Previous participants of mine talked about the idea of being the “bad bitch,” the girl who does not care about having a boyfriend or what anyone thinks about her and is more concerned with herself. Have you felt like you could take on this “bad bitch energy?”
  - *[If yes]* Please describe what that looks like for you.
  - *[If no]* What do you think keeps you from using this “bad bitch energy”? How would your partner react?
10. Given everything we have discussed so far, I am interested in whether you have something else to add.
